# The construction of a TCM knowledge graph and application of potential knowledge discovery in diabetic kidney disease by integrating diagnosis and treatment guidelines and real-world clinical data

**DOI:** 10.3389/fphar.2023.1147677

**Published:** 2023-05-31

**Authors:** Xiaoliang Zhao, Yifei Wang, Penghui Li, Julia Xu, Yao Sun, Moyan Qiu, Guoming Pang, Tiancai Wen

**Affiliations:** ^1^ Wangjing Hospital, China Academy of Chinese Medical Sciences, Beijing, China; ^2^ Kaifeng Hospital of Traditional Chinese Medicine, Henan, China; ^3^ The University of Melbourne, Melbourne, SA, Australia; ^4^ Institute of Information on Traditional Chinese Medicine, China Academy of Chinese Medical Science, Beijing, China; ^5^ Data Center of Traditional Chinese Medicine, China Academy of Chinese Medical Sciences, Beijing, China

**Keywords:** diabetic kidney disease, knowledge graph, multidimensional relationship, hierarchical weight, systematic combing, profound knowledge discovery

## Abstract

**Background:** The complexity and rapid progression of lesions in diabetic kidney disease pose significant challenges for clinical diagnosis and treatment. The advantages of Traditional Chinese Medicine (TCM) in diagnosing and treating this condition have gradually become evident. However, due to the disease’s complexity and the individualized approach to diagnosis and treatment in Traditional Chinese Medicine, Traditional Chinese Medicine guidelines have limitations in guiding the treatment of diabetic kidney disease. Most medical knowledge is currently stored in the process of recording medical records, which hinders the understanding of diseases and the acquisition of diagnostic and treatment knowledge among young doctors. Consequently, there is a lack of sufficient clinical knowledge to support the diagnosis and treatment of diabetic kidney disease in Traditional Chinese Medicine.

**Objective:** To build a comprehensive knowledge graph for the diagnosis and treatment of diabetic kidney disease in Traditional Chinese Medicine, utilizing clinical guidelines, consensus, and real-world clinical data. On this basis, the knowledge of Traditional Chinese Medicine diagnosis and treatment of diabetic kidney disease was systematically combed and mined.

**Methods:** Normative guideline data and actual medical records were used to construct a knowledge graph of Traditional Chinese Medicine diagnosis and treatment for diabetic kidney disease and the results obtained by data mining techniques enrich the relational attributes. Neo4j graph database was used for knowledge storage, visual knowledge display, and semantic query. Utilizing multi-dimensional relations with hierarchical weights as the core, a reverse retrieval verification process is conducted to address the critical problems of diagnosis and treatment put forward by experts.

**Results:** 903 nodes and 1670 relationships were constructed under nine concepts and 20 relationships. Preliminarily a knowledge graph for Traditional Chinese Medicine diagnosis and treatment of diabetic kidney disease was constructed. Based on the multi-dimensional relationships, the diagnosis and treatment questions proposed by experts were validated through multi-hop queries of the graphs. The results were confirmed by experts and showed good outcomes.

**Conclusion:** This study systematically combed the Traditional Chinese Medicine diagnosis and treatment knowledge of diabetic kidney disease by constructing the knowledge graph. Furthermore, it effectively solved the problem of “knowledge island”. Through visual display and semantic retrieval, the discovery and sharing of diagnosis and treatment knowledge of diabetic kidney disease were realized.

## 1 Introduction

Diabetic kidney disease (DKD) is a common microvascular complication of diabetes mellitus (DM) (American Diabetes Association, 2020; Microvascular Complications Group of the Chinese Medical Association Diabetes Branch, 2021). It is a chronic kidney disease (CKD) caused by diabetes, characterized by a high incidence and rapid progression. DKD is currently recognized as the leading cause of CKD and end-stage renal disease (ESRD) (United States Renal Data System | USRDS | NIDDK, 2023). Studies have demonstrated that DKD can affect multiple systems and organs in the body, increasing the risk of cardiovascular disease and all-cause mortality (Zhang et al., 2009; Liang et al., 2017). Traditional Chinese medicine (TCM) diagnoses and treats DKD through syndrome differentiation and treatment, showing high efficacy in relieving clinical symptoms, delaying DKD progression, improving renal function, and enhancing patients’ quality of life. The advantages of TCM’s multi-target and multi-pathway characteristics in DKD treatment have also become increasingly prominent (Lin et al., 2020). Therefore, the mining and comb of TCM knowledge for DKD diagnosis and treatment, as well as the construction of a knowledge graph, are of great significance in guiding the clinical prevention of DKD, controlling disease progression, and improving prognosis.

The knowledge graph is an emerging technology for large-scale knowledge management and intelligent services in the era of big data. It serves as a technical method to describe knowledge and model the association relationships between various entities in the world using graph models (Singhal A, 2012). Knowledge graphs possess key characteristics such as knowledge semantics, easy data association, and expandable data resources (Xiao et al., 2021). And knowledge graphs gather and organize scattered knowledge based on the perspective of “association relationship,” facilitating the integration and fusion of knowledge resources. Knowledge graphs provide an ideal solution to the challenge of “knowledge islands.” In recent years, knowledge graphs have found wide applications in the medical field (Yu et al., 2017; Li X. et al., 2020; Huang et al., 2021; Santos et al., 2022). They offer essential technical means to integrate, share, and deeply explore relevant knowledge on disease diagnosis and treatment. However, most research in this area tends to focus on technical methods, such as optimizing and embedding algorithms like Named Entity Recognition and Disambiguation (Ma et al., 2021; Li et al., 2022; Kurbatova and Swiers, 2021; Qi et al., 2021). There is still a limited number of studies addressing practical clinical problems using knowledge graph technology, and specific challenges remain in the current research on medical knowledge graphs.

1) The source of knowledge is ex parte.

The knowledge sources of existing studies are mostly based on authoritative data such as guideline literature (Yu et al., 2017; Rossanez et al., 2020; Zhu et al., 2020; Huang et al., 2021), or on electronic medical record data (Li L. et al., 2020). While constructing a knowledge graph based on authoritative data, like guidelines, can guarantee data accuracy, the complexity of clinical diagnosis arises from substantial variations among individual patients in the real world. Individual patient factors, including diet, mood, and genetics, can influence doctors’ medication choices, making sole reliance on guidelines unsuitable for all patients and potentially impacting treatment efficacy (Li L. et al., 2020). Additionally, the iterative updating of knowledge lags behind. With the development of medical informatization, electronic medical records (EMR) have been widely popularized (Cheng et al., 2013). EMRs document the entire diagnosis and treatment process, encompassing disease progression, examination results, and medical records. EMR data provides a comprehensive and accurate picture, including comorbidities, confounding factors, and the unique aspects that define individuals. However, EMR data also shows the problem of confusing data structure and the need for more authority. Therefore, an accurate grasp of data sources is crucial for acquiring information.

2) Data entity and relationships are embodied as coroughse granularity.

The medical knowledge graph primarily relies on ontology for construction, which involves defining classes, relationships, and attributes within the graph. Ontology construction mainly revolves around reusing existing ontologies in the field. The data layer supplements the ontology layer by extracting entities and relationships from the original data. However, this approach leads to a coarse granularity of the conceptual and relational data obtained, potentially resulting in a relatively weak clinical guidance effect. Clinical data still contains a significant amount of implicit information, and organizing, mining, and analyzing the data can enrich the entities, attributes, and relationships, allowing for the expansion of knowledge into more refined granularity.

Although research on the rules for TCM diagnosis and treatment of DKD has increased in recent years, most previous studies have relied on data mining technology to analyze the diagnosis and treatment rules of DKD from a limited perspective (Wang et al., 2014; Li et al., 2016; Li et al., 2019). The acquisition of DKD knowledge has been relatively one-sided and has not formed a comprehensive knowledge system, which has contributed to the delayed progress in research on TCM diagnosis and treatment of DKD. This paper integrated DKD’s TCM diagnosis and treatment guidelines, consensus, and actual medical records data. The previous data mining results are introduced into the construction of DKD TCM diagnosis and treatment knowledge graph, and the relationships in the map are given corresponding weights and levels. The construction of DKD knowledge graph of TCM diagnosis and treatment makes future DKD knowledge reasoning research possible. The knowledge graph can make the acquisition of DKD diagnosis and treatment knowledge more intuitive and convenient. Through the retrieval of the relationship between diagnosis and treatment, we can further discover the tacit knowledge of DKD in TCM diagnosis and treatment, and continuously iterate and update the TCM diagnosis and treatment knowledge of DKD.

## 2 Materials and methods

### 2.1 Overview of the research process

**FIGURE 1:**
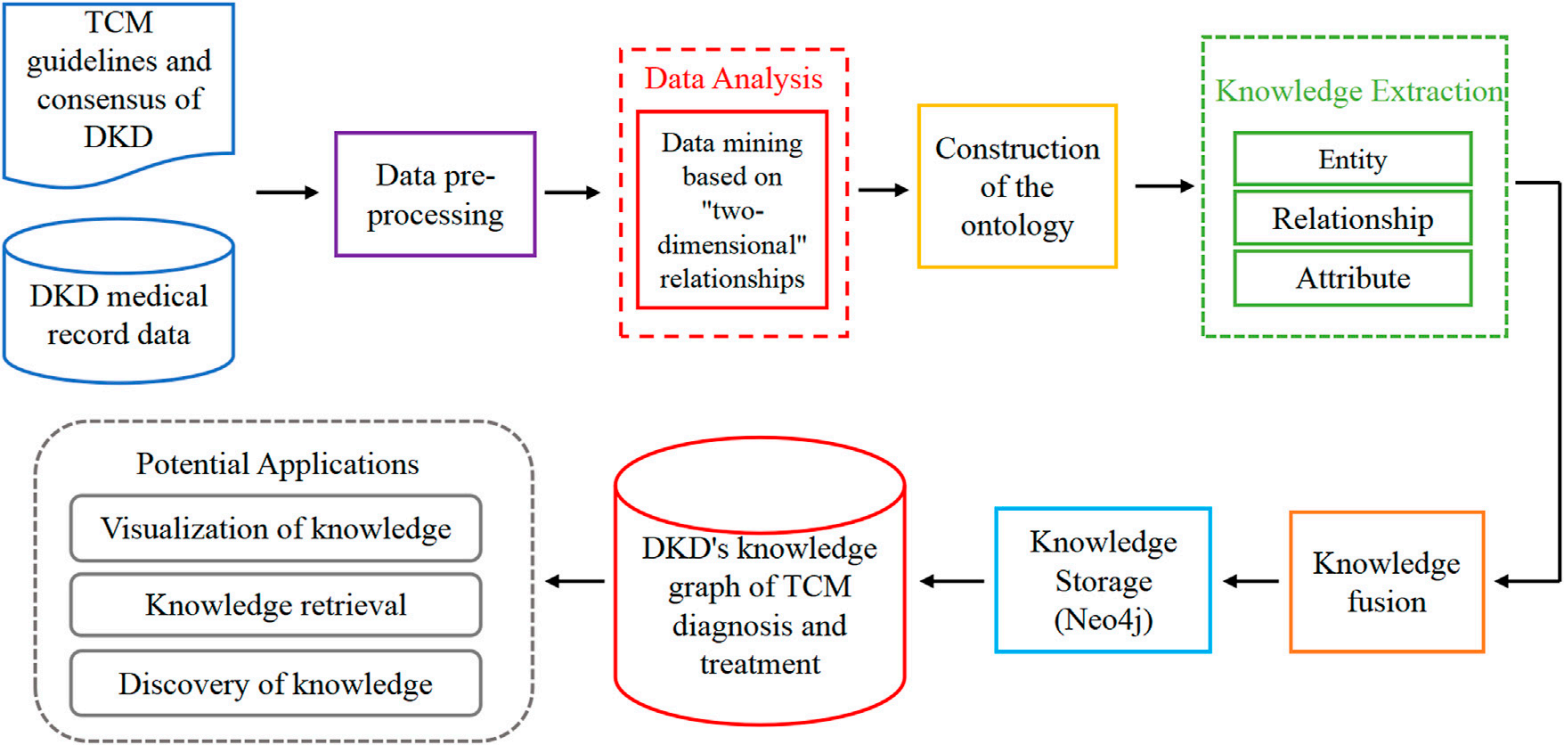
The construction and application process of TCM diagnosis and treatment knowledge graph of DKD. The main method is based on ontology. Specific steps include data preprocessing, data analysis, construction of the ontology layer, knowledge extraction, Knowledge fusion, and knowledge storage. After the construction of the graph, it is applied to knowledge visualization, knowledge retrieval, and discovery.

The objective of this study is to develop an authoritative and practical knowledge graph of TCM diagnosis and treatment for DKD, and facilitate the in-depth discovery of knowledge based on this framework. The construction process of the DKD TCM diagnosis and treatment knowledge graph primarily adopts an ontology-based construction method. The steps involved in this process include data preprocessing, data analysis, ontology construction, knowledge extraction, knowledge fusion, and knowledge storage. Once the graph construction is completed, it can be applied to knowledge visualization, knowledge retrieval, and knowledge discovery (Figure 1). In contrast to the traditional approach to knowledge graph construction, this study initially conducts data mining on the original data to enhance the accuracy and interpretability of the knowledge graph. Subsequently, the study mines and analyzes the implicit knowledge within the data based on the two-dimensional relationship. Therefore, the input sources incorporated in the graph construction mainly encompass the following:

1) High-quality data on TCM diagnosis and treatment guidelines for DKD.
2) Clinical medical record data of DKD in TCM.
3) Relational data obtained through data mining.

### 2.2 Data sources

The data sources of this study mainly include two aspects: 1) Guidelines, expert consensus, standards, and protocols related to TCM diagnosis and treatment of diabetic kidney disease. These include “*Guidelines for the diagnosis and treatment of DKD combined with disease and syndrome (2022)*" (Yu et al., 2022), “*TCM diagnosis and treatment plan for wasting-thirst kidney disease (diabetic kidney disease) (2017)*" (Dongzhimen Hospital of Beijing University of Traditional Chinese Medicine, 2017), “*TCM diagnosis and treatment standards for DKD (2011)*" (Gao et al., 2011), and “*DKD diagnosis, syndrome differentiation, and efficacy evaluation standards (2007)*." (Yang and Liu, 2007). 2) Effective inpatient medical records were obtained from DKD patients hospitalized at Kaifeng Hospital of TCM from December 2018 - June 2021 (A total of 1105 cases were included). The medical record data in this study contains the essential characteristics, symptom description (including the tongue, pulse, urine and stool), treatment principles and methods, prescription names, auxiliary examination results, and curative effect evaluation statements of DKD patients. The medical record data include disease diagnosis (Western medicine diagnosis, complications, comorbidities), TCM syndrome diagnosis and prescribed medication.

### 2.3 Data preprocessing

#### 2.3.1 Data screening

The data included in this research were selected through a combination of primary database screening and manual secondary screening. The specific screening criteria were 1) a confirmed diagnosis of type 2 diabetic kidney disease. 2) Age ≥18 years old. 3) complete set of crucial information in patients’ data and medical records was complete. (The medical record information includes TCM diagnosis, western medicine diagnosis, staging diagnosis, syndrome diagnosis, chief complaint, current symptoms, etc. The laboratory examination information comprises at least two examinations on admission and discharge). 4) Information related to efficacy evaluation is available (after treatment, the main symptoms of DKD have improved significantly, relevant indicators have improved or returned to normal, and “obvious remission,” “obvious relief,” “obvious improvement,” “disappearance,” etc. appear in the description of discharge summary).

#### 2.3.2 Efficacy evaluation analysis of data

As this study is retrospective, the included data are mainly medical records of real-world TCM diagnosis and treatment of DKD. To reduce bias caused by confounding factors and improve the accuracy and clinical applicability of atlas discovery knowledge. This study evaluated the efficacy of the included data through primary database screening and manual secondary screening validation methods. Concerning the “DKD diagnosis, syndrome differentiation, and efficacy evaluation standards (2007).” (Yang and Liu, 2007); “Studies about Specification of Syndrome Differentiation on Different Stages and Efficacy Evaluation Proposal for Diabetic Kidney Disease. (2017).” (Zhao et al., 2017), in combination with the data included in this study and the suggestions of experts in the field, the efficacy evaluation indicators of retrospective medical record data in this study were formulated (Table 1). Finally, 1105 DKD patients’ TCM diagnosis and treatment data were included.

**TABLE 1:** Efficacy evaluation indicators of DKD.

Evaluation indicators	Content of the evaluation description	Evaluation description
Main symptoms	edema、foamy urine、lack of strength、shortness of breath、dry mouth、thirst、constipation、numbness in limbs、positioning sting、soreness and weakness of the waist and knees, etc	improve、relieve、relief、vanish、ameliorate、inapparent、no recurrence、recovery. etc.,
Urine-related indicators	mALB、24hUTP、UACR、UAER	decrease or return to normal
Blood-related indicators	GLU、HbA1c、SCr	decrease

#### 2.3.3 Data standardization

Targeting the problems of irregular use of terminologies, confusion of concepts, and clutter of terminology information in the data included in this study, the data standardization process can normalize, organize and improve the quality of the data. In this study, the Western medical diagnosis was standardized with reference to the *International Classification of Diseases-11 (ICD-11) (* ICD-11, 2023). The specification on the syndrome names was taken from “*Clinic terminology of traditional Chinese medical diagnosis and treatment—Part 2: syndromes*” (National Health Commission, State Administration of traditional Chinese medicine, 2020a). The classification standard of syndrome elements in TCM was guided by “*syndrome differentiation of syndrome elements*" (Zhu, 2008). The data of symptoms and signs were standardized with reference to *the “Standardized Terminology of Common Clinical Symptoms in TCM"* (Li and Ma, 2015), *“Differential Diagnosis of Symptoms in TCM”* (Yao, 2005), and *“Terminology of TCM”* (Committee for the examination and approval of terms in traditional Chinese medicine, 2005). The specification of the therapeutic methods was taken from *“Clinic terminology of traditional Chinese medical diagnosis and treatment—Part 3: therapeutic methods*” (National Health Commission, State Administration of traditional Chinese medicine, 2020b). Finally, the names of herbal medicines extracted from medical prescriptions were classified according to *“the Pharmacopoeia of the People’s Republic of China (2020 edition)”* (National Pharmacopoeia Committee, 2020). Data standardization included categorization of upper-level terminology, unification of synonymous terms, filling of referential terms, and simplification of redundant terms (Table 2).

**TABLE 2:** Example of data standardization.

Number	Standardized methods	Original terminology	Standardized terminology
1	Categorize to superior terms	Grade III hypertension, Hypertension Grade 3 (high risk)	Hypertension
2	Harmonization of synonymous terms	Xianlingpi, Epimedium	Epimedium
3	Normalization of the expression of irregular terms	Syndrome of intense exuberant fire toxin	Syndrome of intense exuberant heat toxin
4	Filling of pronouns	Numbness and tingling in both lower limbs	Numbness in the lower limbs, tingling in the lower limbs
5	Simplification of redundant terms	Itching of both feet	Itchy feet

### 2.4 Data analysis

**FIGURE 2:**
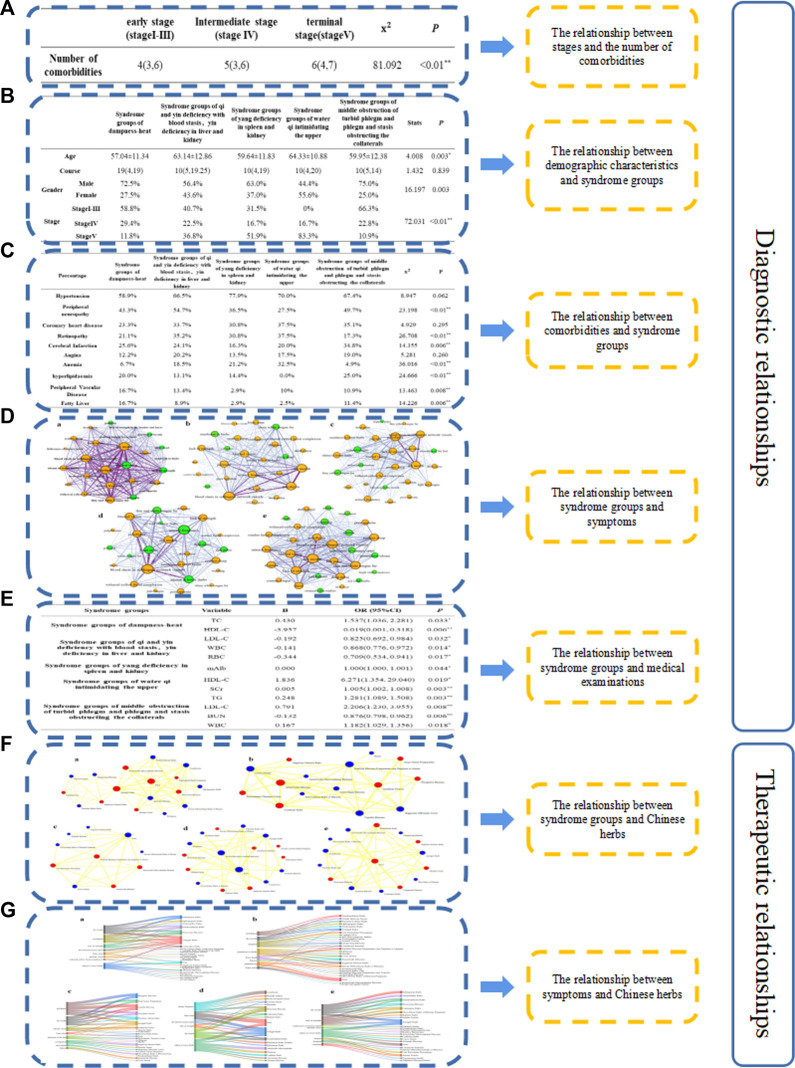
Process of two-dimensional relational mining and combing. **(A)** The relationship between stages and the number of comorbidities **(B)**. The relationship between “demographic characteristics and syndrome groups”. **(C)** the relationship between comorbidities and syndrome groups. **(D)** The relationship between syndrome groups and symptoms. Different subgraphs represent different syndrome groups, nodes represent symptoms, the size of nodes represents the frequency, green nodes are characteristics symptoms under the syndrome, and edges represents the weighting between two nodes **(E)**. The relationship between syndrome groups and medical examinations. **(F)** The relationship between syndrome groups and Chinese herbs. Different subgraphs represent core prescriptions under various syndromes, nodes represent Chinese herbs, the size of nodes represents the frequency of Chinese herbs, and the edges between nodes represents the weight of drug co-occurrence. **(G)** Different sub-graphs represent different syndrome groups. The left node represents symptoms, and the right node represents Chinese herbs. Edges represents flow data between Chinese herbs and symptoms. The wider the edge, the larger the value. (*p* < 0.05 indicates a statistically significant difference. **(A)** is the syndrome group of qi and yin deficiency with blood stasis, yin deficiency in liver and kidney. **(B)** is the syndrome group of middle obstruction of turbid phlegm, phlegm and stasis obstructing the collaterals; **(C)** is the syndrome group of dampness-heat; **(D)** is the syndrome group of Yang deficiency in spleen and kidney; **(E)** is the syndrome group of water qi intimidating the upper).

In order to obtain Fine Granularity objects and relationships in the process of DKD TCM diagnosis and treatment, and more comprehensive reflect the knowledge of DKD TCM diagnosis and treatment, this study conducted two-dimensional correlation knowledge mining from the perspective of “stage-number of comorbidities”, “comorbidity-syndrome groups”, “demographic characteristics-syndrome”, “syndrome groups-typical symptom”, “syndrome groups -medical examination”, “syndrome groups-Chinese herb”, and “symptom-Chinese herb” (Figure 2). According to the frequency distribution, corresponding weights were given to the relationships, and the corresponding levels were divided. Mining the relationships among entities can enrich the relational data in the knowledge graph and provide a foundation for organizing and discovering subsequent knowledge. Through two-dimensional relational data mining, we obtained 79 correlations, associations, and treatment relationships (Table 3).

**TABLE 3:** Relationships from data mining.

Number	Relationships	Type of relationships	Number of relationships
1	Stage-Number of comorbidities	correlation	1
2	Comorbidities-Syndrome groups	correlation	7
3	demographic characteristics-syndrome groups	correlation	3
4	syndrome groups- typical symptoms	contain	5
5	syndrome groups- medical examinations	correlation	12
6	syndrome groups-Chinese herbs (core prescriptions)	treat	5
7	Symptoms-Chinese herbs	treat	38

### 2.5 Construction of ontology

The DKD TCM diagnosis and treatment knowledge graph consists of a pattern layer and a data layer. The pattern layer, also known as the ontology layer, represents the theoretical aspect of the knowledge graph and defines its high-level structure. The data layer, on the other hand, encompasses the specific instances related to diagnosis and treatment, serving as a concrete manifestation of the pattern layer. The goal of ontology construction is to acquire, describe, and represent domain knowledge, establish a shared understanding of the domain, identify commonly accepted terms, and provide clear definitions for these terms and their interrelationships across different levels of formal patterns (Liao, 2007; Guarino et al., 2009). The construction methods of knowledge graph technology can be categorized into two types: top-down and bottom-up. The “top-down” approach involves defining a schema layer (ontology) based on the logical relationships and hierarchical structure of knowledge, and subsequently mapping data entities to this schema. On the other hand, the “bottom-up” approach entails extracting entities and attributes from diverse data sources into the data layer of the knowledge graph, consolidating the extracted entities and attributes, and optimizing the schema layer of the knowledge graph to facilitate iterative updates of the ontology model (Wang et al., 2019). The top-down method ensures the professionalism and accuracy of the constructed domain ontology, while the bottom-up method ensures its practicality. Given the unique characteristics of medicine, this study employs both top-down and bottom-up methods to construct a DKD TCM diagnosis and treatment knowledge graph, aiming to enhance its guidance, accuracy, and practicality.

**FIGURE 3:**
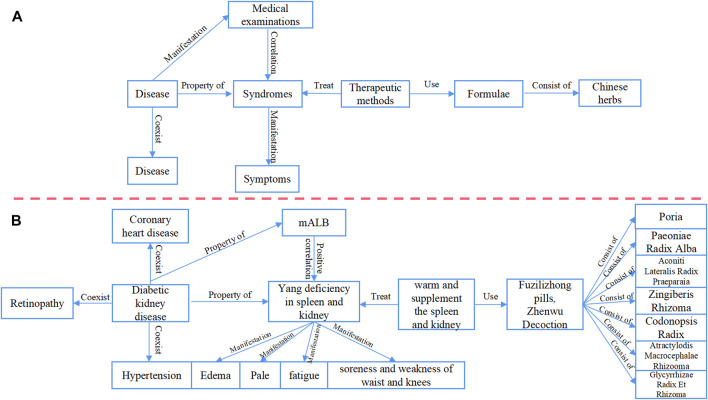
Logical framework of the pattern layer and data layer **(A)**. Pattern layer **(B)**. Data layer.

During the process of data screening, standardization, and analysis, this study has conducted initial organization and standardization of DKD TCM diagnosis and treatment knowledge. To achieve this, the study has leveraged the existing Chinese Medicine Language System (TCMLS) (ISO, 2014) for defining concepts and semantic relations, thus collaboratively building the ontology layer. The data layer aims to construct a comprehensive knowledge graph and facilitate the systematic categorization and exploration of DKD TCM diagnosis and treatment knowledge. This is achieved by mapping information and knowledge into concepts and relationships constructed in the pattern layer using a triple format of “entity-relationship-entity” and “entity-attribute-attribute value.” (Figure 3).

### 2.6 Knowledge extraction

The data in this study comprise unstructured data such as guidelines and consensus, as well as semi-structured and structured data from real-world clinical records. The data structure was complex, and the unstructured part of the medical records often contained pronouns and omissions in the language. Recently, methods like named entity recognition have been widely used to extract entities and relationships. However, automated systems are generally better at identifying sentences with common relationships, while they struggle to extract complex relationships and may miss less prominent relationships that are known. Given the complexity of DKD data in TCM diagnosis and treatment and the aim to ensure the accuracy of the knowledge graph, this study adopts an interactive approach where two TCM practitioners manually extract entities, relationships, and attributes, and organize them as triplets.

If there is a disagreement between the two TCM practitioners, a third senior TCM practitioner is invited to evaluate the extracted knowledge. This study takes concepts and the relationship between concepts as the extraction framework. Since the incorporated data contains data mining results, there are regular relationships with hierarchy and weight in relation extraction (hierarchy and weight are mainly determined by frequency). Therefore, it is important to take this fully into account when defining the extraction framework, filling in definitions of concepts and relationships.

### 2.7 Knowledge storage

This study used the Neo4j graph database for knowledge storage. The storage principle of this graph database is to use nodes, edges, and attributes to store graph data. Neo4j is currently the most popular high-performance NoSQL graph database (Gong et al., 2018), with features of high availability, stability, scalability, and strong intuitiveness. This study predominantly used the LOAD CSV statement of Cypher language to import data which allows the loading of data locally or remotely to import CSV files for real-time insertion, which is convenient for future data addition, deletion, and updating.

### 2.8 Potential applications of knowledge graphs

Knowledge graphs have found extensive application in the medical field. This study integrates knowledge graphs with expert experience to investigate the potential directions for knowledge discovery through human-computer collaboration. TCM is characterized by its experiential nature, with diagnostic and treatment knowledge predominantly derived from doctors’ clinical experience, which is highly subjective. Consequently, this study assesses the performance of the DKD TCM diagnosis and treatment knowledge graph by employing reverse validation based on expert diagnostic and treatment reasoning. Firstly, the Connected Components algorithm retrieves the multi-level correlation of different kinds of knowledge. At the same time, TCM experts in the field of nephrology and endocrinology are invited to elaborate on the critical links in the diagnosis and treatment of DKD in TCM and raise questions. Afterward, a Cypher statement was used to verify whether the results obtained from the DKD TCM knowledge graph met the experts’ expectations and whether it could achieve the sorting of diagnosis and treatment knowledge and the knowledge discovery of implicit treatment thinking.

After communication with clinical experts, the following five questions were summarized for knowledge retrieval.

1) There are many kinds of syndrome diagnoses in the process of TCM diagnosis of DKD. Can the knowledge graph reflect the coexistence of multiple syndromes (deficiency-excess complex) in TCM diagnosis of DKD?
2) Does it reflect the addition and subtraction of drugs for specific symptoms?
3) Is there a situation where a single Chinese herb treats different symptoms during drug addition and subtraction?
4) Does the selection of treatment plans vary based on patients with different underlying diseases?
5) Are there any differences in treatment options for the same syndrome diagnosis at different stages?

## 3 Results

### 3.1 The concept and type of DKD TCM diagnosis and treatment ontology

In this study, a total of nine concepts and types in the ontology were defined using DKD’s TCM diagnosis and treatment guidelines, expert consensus, and real-world medical record data, including diseases, stages, syndrome groups, syndromes, medical examinations, symptoms, Chinese herbs, therapeutic methods, and formulae. In addition to the syndrome groups derived from real-world data, the other eight concepts were derived from guidelines and consensus (Table 4).

**TABLE 4:** Sources and definitions of ontology concepts.

No.	Concept terms	Source	Meaning
1	Diseases	Guidelines, expert consensus, standards, schemes	Under the infection of the six pathogenic factors, the seven emotions, and other pathogenic factors, the internal and external environment of the body is out of balance, and then behavior and mental changes occur. It is manifested as certain symptoms and signs of pathological processes
2	Stages	Guidelines, expert consensus, standards, schemes	Diagnosis of disease severity and status
3	Syndrome groups	Real-world medical record data	In the disease process of syndrome, when one syndrome element occurs, several other syndrome elements may occur concurrently. These syndrome elements are combined to form syndrome groups
4	Syndromes	Guidelines, expert consensus, standards, schemes	The syndrome is the external presentation of the reaction status of the essential organ connection of the disease location, etiology, disease nature, disease trend, and the strength of the body’s resistance in a particular stage of the disease process, clinically observable symptoms are shown
5	Symptoms	Guidelines, expert consensus, standards, schemes	Symptoms are presentation of the abnormal status of the organ, which include various abnormal sensations felt by the patients themselves, and various abnormal manifestations sensed by the doctors
6	Medical examinations	Guidelines, expert consensus, standards, schemes	Medical examination refers to the physical or chemical test carried out in the laboratory to determine the content, properties, concentration, quantity, and other characteristics of the substances submitted for examination
7	Therapeutic methods	Guidelines, expert consensus, standards, schemes	The therapeutic method refers to the specific method of treating disease guided by the treatment principles
8	Formulae	Guidelines, expert consensus, standards, schemes	A formula is a Chinese medicine prescription composed of herbs according to the principles of compounding guidelines and the clinical experience of doctors
9	Chinese herbs	Guidelines, expert consensus, standards, schemes	Chinese herbs are natural products used for prevention, treatment, and healthcare under the guidance of TCM theory and clinical experience, including raw herbs and decoction pieces

### 3.2 Semantic relationship between concepts of DKD TCM diagnosis and treatment

We defined a total of nine semantic relations (object properties) between concepts, including process, consisting of^…^, treat, coexist, use, contain, manifestation, property of, and correlation. Among them, the process, contain, manifestation, treat, use and consisting of were derived from guidelines and consensus. Combined with the analysis results of real medical record data, the above relationships were supplemented, and three relationships of coexist, property of and correlation were added (Table 5).

**TABLE 5:** Sources and definitions of semantic relations.

No.	Relation	Source	Meaning
1	Process	Guidelines, expert consensus, standards, schemes	Behavior, state, or function
2	Consist of	Guidelines, expert consensus, standards, schemes	It is used to describe the relationship between the whole and the individual. It is relative to macro description and expresses macro imagery based on segmentation
3	Treat	Guidelines, expert consensus, standards, schemes	Treatment is usually the process of intervening or changing a specific health condition. It is a process in which a means or measure acts on the human body
4	Coexist	Real-world medical record data	It is the process by which one event or activity causes another event or activity. This study refers to the process by which a pre-existing disease causes another disease
5	Use	Guidelines, expert consensus, standards, schemes	It refers to the service of instructing personnel, utilizing utensils, or funds to serve a purpose. In this study, it mainly refers to the process of using certain means or methods to treat disease
6	Contain	Guidelines, expert consensus, standards, schemes	It is the relationship between a set and a collection, also called the subset relationship. It means the existence of one thing in another
7	Manifestation	Guidelines, expert consensus, standards, schemes	The external form of things in development and change. It refers to all the situations where things are shown and felt by people
8	Property of	Real-world medical record data	A property that is unique to something, peculiar to that thing
9	Correlation	Real-world medical record data	It means that two things are related to each other. That is a shared relationship or causal relationship between things or signals

**FIGURE 4:**
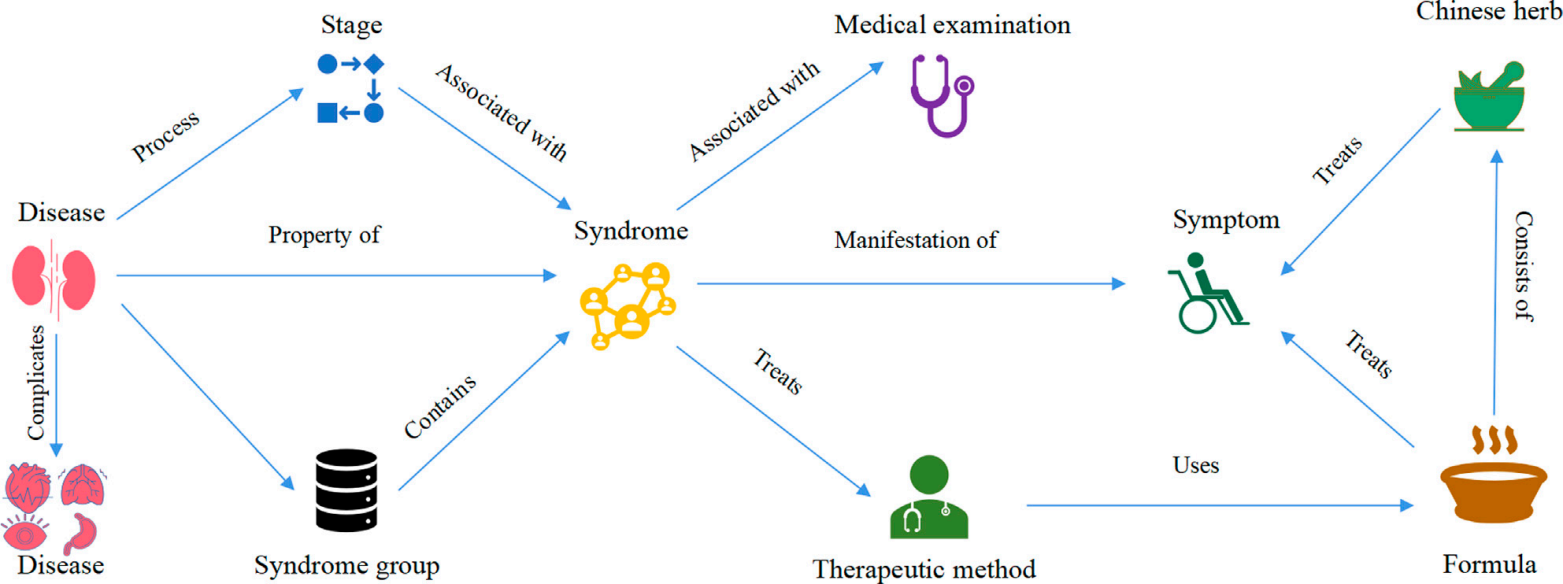
Ontology layer construction DKD TCM diagnosis and treatment knowledge graph.

In order to better express the strength of the relationship between entities and to have a clearer understanding of the knowledge of DKD TCM diagnosis and treatment, based on the frequency of the two-dimensional relationships between entities, we gave weights to the prominent relationships and divided them into grades. Ultimately, this study resulted in an ontological framework containing nine concepts and 20 relationships (Figure 4).

### 3.3 DKD TCM diagnosis and treatment knowledge graph

Using results from previous data mining based on two-dimensional relationships, this study included the division of relationship existence grades and weights (grades and weights are mainly judged by frequency). A standardized manual review of knowledge extraction results was performed to correct errors and deficiencies in the results. At the same time, an EXCEL database was established for the extracted results. This database was then reviewed by two experts in the field, and the rationality and accuracy of the extracted results were re-evaluated from a professional perspective. Finally, a DKD TCM diagnosis and treatment knowledge graph with 903 nodes and 1669 relationships was formed.

**FIGURE 5:**
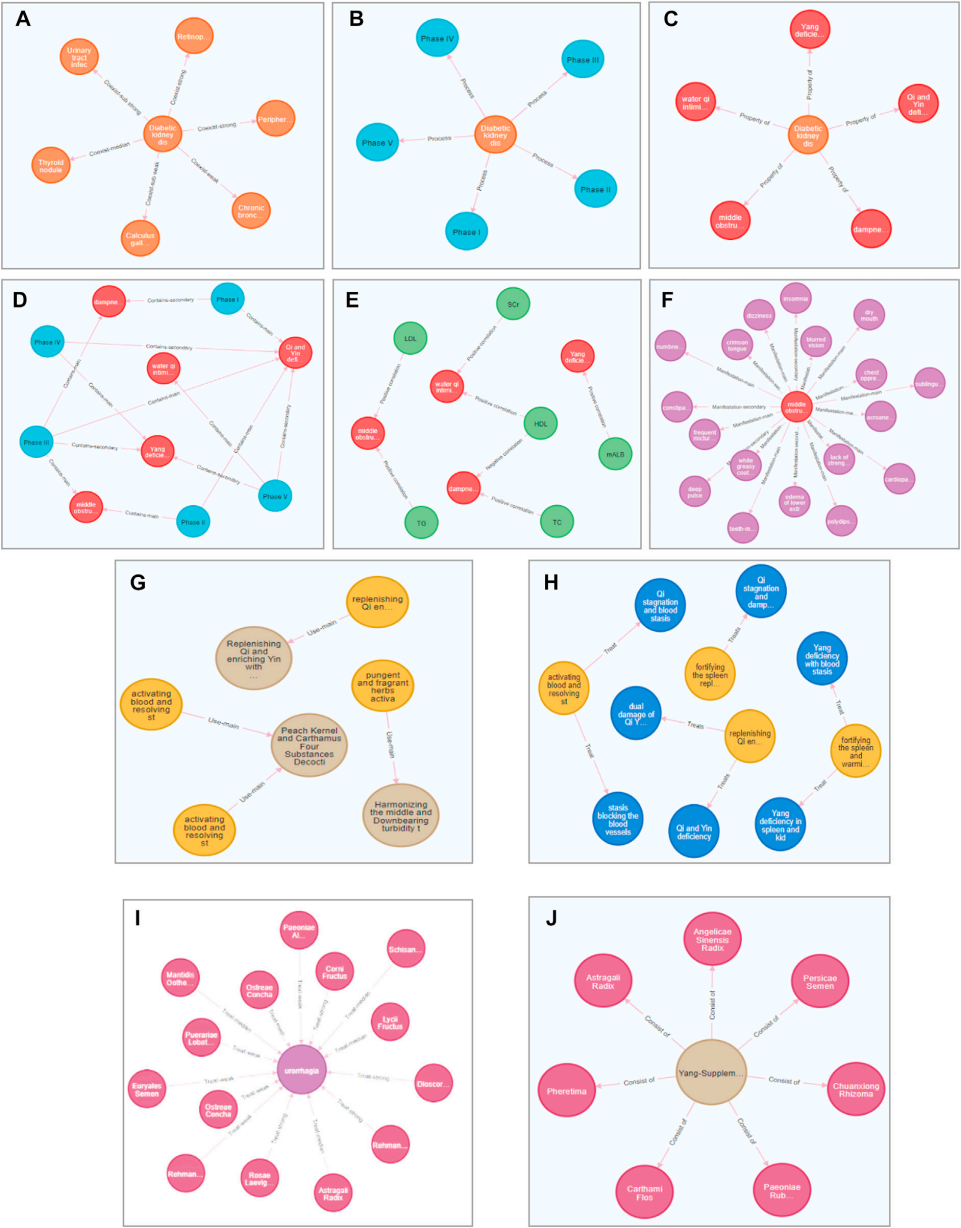
Concept and relatonship distribution of DKD TCM diagnosis and treatment knowledge graph. **(A)** disease-disease. **(B)** disease-stage. **(C)** disease-syndrome groups. **(D)** stage-syndrome groups. **(E)** medical examination-syndrome groups. **(F)**. syndrome group-symptoms. **(G)** therapeutic method-formulae. **(H)** syndrome-therapeutic method. **(I)** Chinese herbs-symptoms. **(J)** formulae-Chinese herbs. (Nodes represent entities, edges represent relationships, and different colors represent different entities. Orange nodes in A, B, and C represent diseases; light blue nodes in B and D represent stages; red nodes in C, D, E, and F represent syndrome group; green nodes in E represent mediical examination; purple nodes in F and I represent symptoms; yellow nodes in G and H represent therapeutic method; brown nodes in G and J represent formulae; dark blue nodes in H represent syndromes; pink nodes in I and J represent Chinese herbs).

In this study, nine conceptual nodes of “disease, stage, syndrome groups, syndromes, medical examination, symptoms, Chinese herbs, and formulas” were included. A total of 10 inter-nodal relationships were formed between “disease-disease”, “disease-stage”, “disease-syndrome groups”, “stage-syndrome groups”, “medical examination-syndrome groups”, “syndrome groups-symptoms”, “therapeutic method-formulae”, “syndrome-therapeutic method”, “Chinese herbs-symptoms”, and “formulae-Chinese herbs" (Figure 5).

### 3.4 Mining and verification of TCM diagnosis and treatment knowledge in DKD based on human-computer interaction

In this study, a knowledge graph of DKD TCM diagnosis and treatment was constructed by integrating guidelines and real-world clinical case data. Drawing upon the “multi-dimensional relationship” and “multi-hop query” characteristics of the knowledge graph, we extracted and organized the implicit knowledge embedded in case data. Through the utilization of the Connected Components community discovery algorithm and Cypher sentence search, we thoroughly explored and analyzed the DKD TCM diagnosis and treatment knowledge graph. Employing a human-computer combination approach guided by experts’ key concerns, we verified the performance of the graph.

#### 3.4.1 Multi-hop hierarchical knowledge discovery of DKD TCM diagnosis and treatment

**FIGURE 6:**
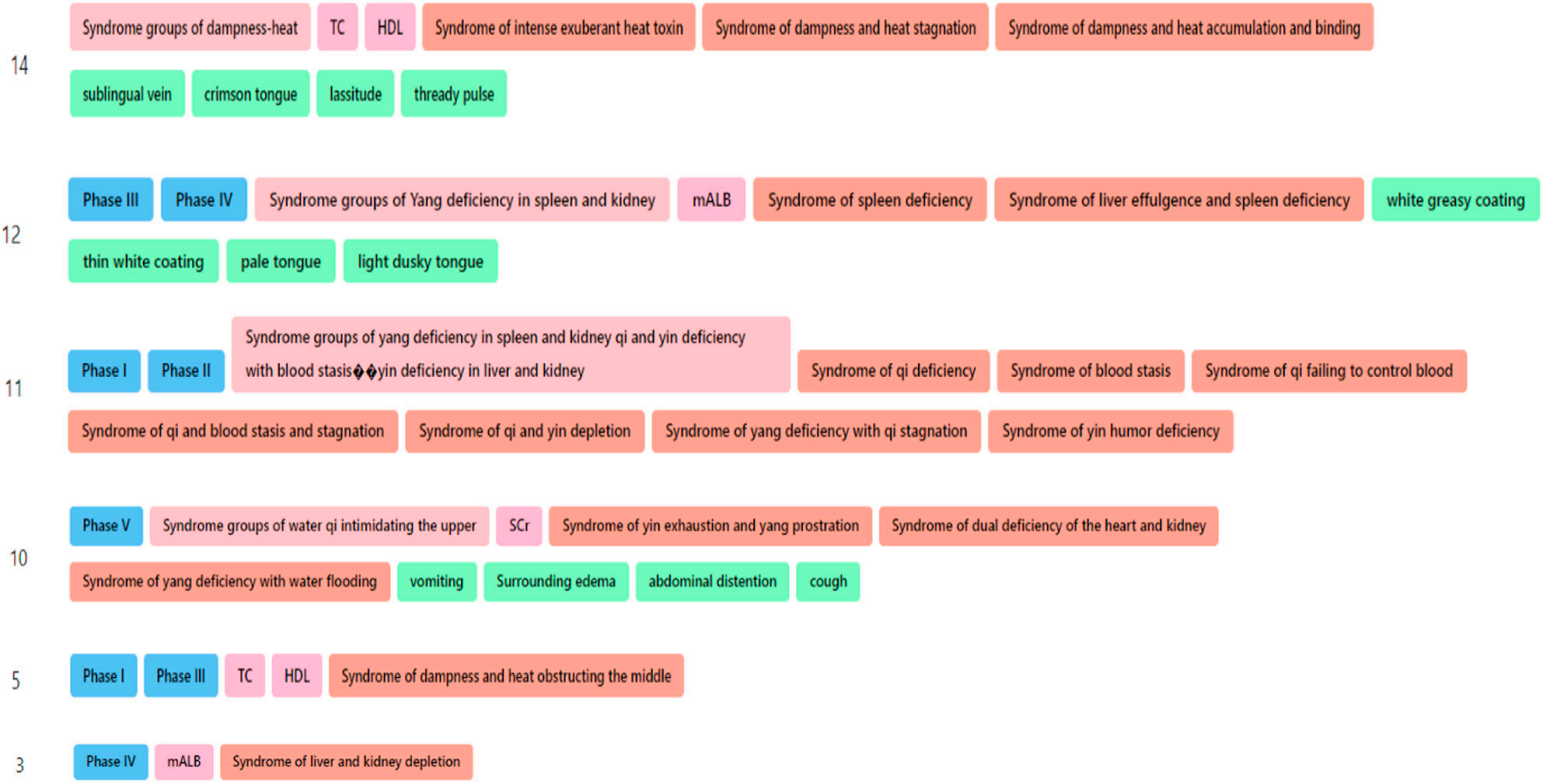
Examples of multi-level knowledge discovered by the Connected Components community. (Nodes with different colors represent different entities).

In this study, the community discovery algorithm of Connected Components was utilized to conduct community clustering analysis on the nodes within the DKD TCM diagnosis and treatment knowledge graph. Through this analysis, the multi-level relationship among stages, syndrome groups, syndromes, laboratory indicators, symptoms, and other entities was established. For instance, patients in DKD stage III and IV were predominantly diagnosed with spleen and kidney yang deficiency syndrome, which was associated with the level of mALB. The main clinical manifestation includes a white greasy coating or thin white coating on the tongue. On the other hand, patients in DKD stage V were mainly diagnosed with the syndrome of water qi intimidating the upper, which showed a close relationship with the SCr level. Additionally, these patients often exhibited symptoms such as vomiting, surrounding edema, abdominal distention, and cough (Figure 6). The results were evaluated by two TCM experts and found to be consistent with clinical practice.

#### 3.4.2 DKD’s TCM syndrome diagnosis of " deficiency-excess complex."

According to experts, there are often many kinds of syndrome diagnosis in the DKD diagnosis and treatment process, that is, the situation of the deficiency-excess complex. This study conducted a search using Cypher sentences, which are as follows:

MATCH (t:`Therapeutic method`)-[r:Treats]->(s:Syndrome), (m:`Syndrome groups`)-[r2:Contains]->(s:Syndrome)

WHERE t. name = ‘replenishing qi and enriching yin, activating blood and resolving stasis’.

RETURN m,t,s,r,r2.

**FIGURE 7:**
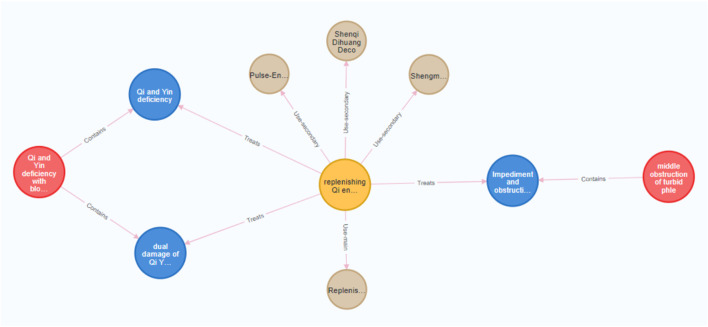
Examples of “deficiency-excess complex” in Diagnosis of DKD Traditional Chinese Medicine Syndrome. (Red nodes represent the syndrome groups. blue nodes represent the syndromes, the yellow node represent the therapeutic method. And brown nodes represent formulae).

The results revealed that there were cases where multiple syndromes coexisted, and different syndromes were treated using the same therapeutic approach. For instance, in the diagnosis of the same patient, both the syndrome groups of “Qi and Yin deficiency with blood stasis, liver-kidney Yin deficiency” and “phlegm-turbid obstruction, phlegm-blood stasis blocking collateral” were identified. Furthermore, all of these syndromes were treated with the method of benefiting Qi and nourishing Yin, combined with invigorating blood and resolving stasis (Figure 7). This finding aligns with the concept of the “deficiency-excess complex” proposed by experts. During the progression of the disease, it is common for patients to experience the coexistence of multiple syndromes and a mixture of deficiency and excess.

However, experts have pointed out that relying solely on search results may lead to certain ambiguities and misunderstandings. TCM emphasizes the principle of “treating different diseases with the same treatment” rather than “treating different syndromes with the same treatment.” The treatment process primarily revolves around syndrome differentiation. The knowledge obtained from the graphs indicates that applying the same treatment method for different syndromes is inconsistent with clinical practice. Therefore, clinicians should interpret and assess the knowledge graph in conjunction with their own expertise. In the presented results, different syndromes are identified, but the same therapeutic methods are employed. From the perspective of DKD pathogenesis, the syndromes of DKD exhibit a “deficiency of the root and excess of the symptoms” pattern, where a combination of deficiency and excess may be present in syndromes such as Qi-Yin deficiency, Qi-Yin damage, and blood stagnation and obstruction. In the first two syndromes, Qi-Yin deficiency is considered the primary issue, while blood stasis is secondary. Conversely, the syndrome of blood stagnation and obstruction is primarily characterized by excessive blood stasis, with less pronounced deficiency in both Qi and Yin. This underscores the importance of fully considering the characteristics of individual diseases during clinical syndrome diagnosis. The factors of “deficiency and excess” and “primary and secondary” should be distinguished, and similar considerations should also be taken into account during the treatment process.

#### 3.4.3 Discovery of symptomatic knowledge of addition and subtraction of herbal treatment

TCM treatment has the characteristics of individualization. In addition to the process of syndrome differentiation and treatment, the addition or subtraction of herbs are also considered according to specific symptoms. Clinical addition and subtraction is also key issue in diagnosing and treating DKD in TCM. According to the experts put forward the problem of addition and subtraction medication. In this study, specific Cypher sentences were searched, taking skin itching as an example, and the specific sentences were as follows.

MATCH (c:`Chinese herb`)-[r]-(s:symptom).

WHERE s. name = ‘Itch of skin’ RETURN c, r, s.

**FIGURE 8:**
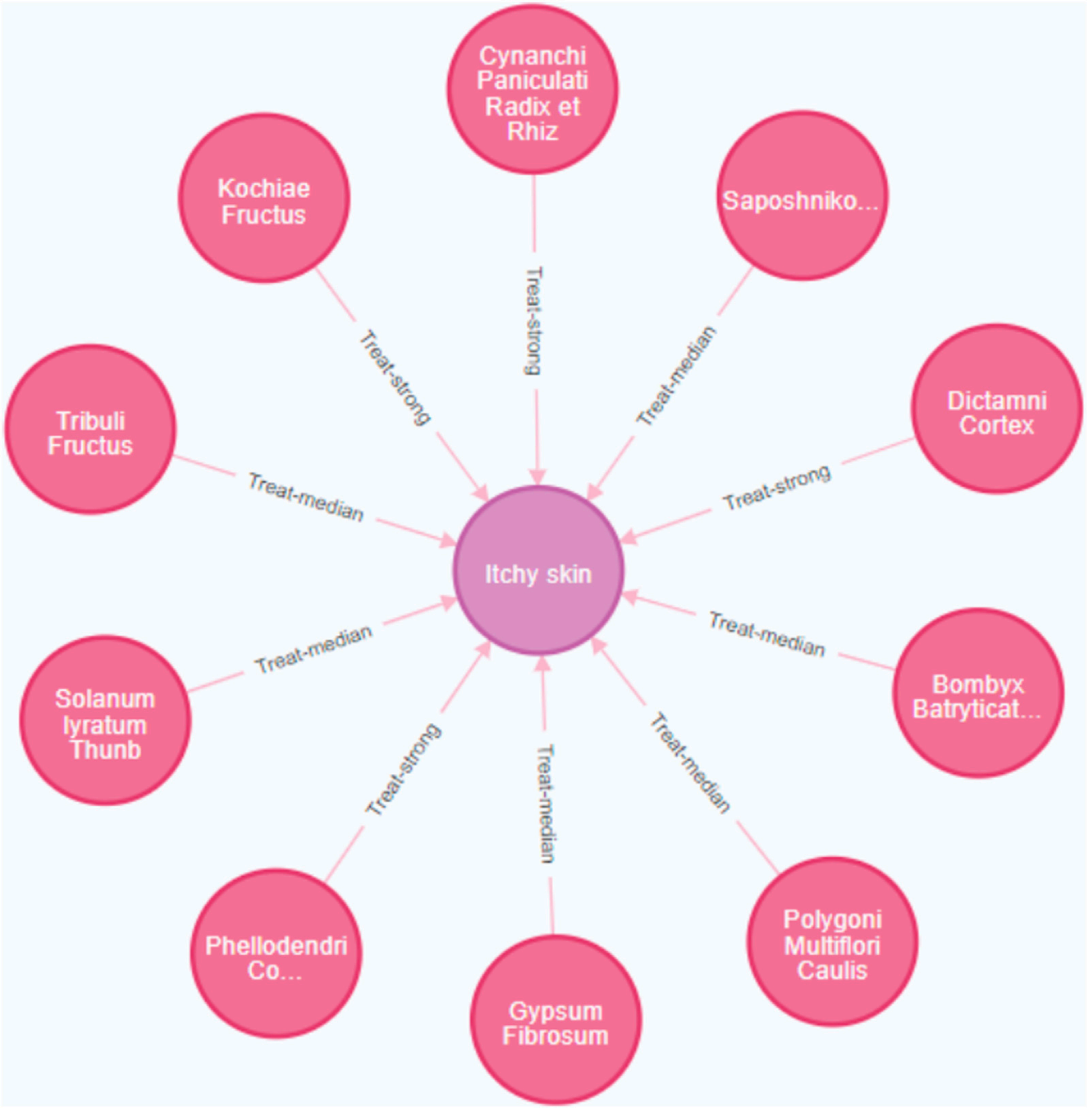
Knowledge discovery of Chinese herbs addition and subtraction. (Pink nodes represent Chines herbs, and the purple node represents the symptom).

Through semantic search, “symptomatic treatment” herbs can be discovered. Reference can be provided for clinical symptomatic herb selection according to the weight prompt. For example, for the symptoms of itchy skin, herbs such as Kochiae Fructus, Radix et Rhizoma Cynanchi Paniculati, Batryticatus Bombyx, Radix Saposhnikoviae, Dictamni Cortex can be selected. However, according to the weight in the DKD knowledge graph, Kochiae Fructus and Dictamni Cortex have a stronger relationship for the treatment of itchy skin. Therefore, these herbs are more recommended in the process of clinical herb selection. However, further consideration of the patient’s specific situation should be given (Figure 8). The retrieved drugs were basically consistent with the routine clinical medication through expert identification.

#### 3.4.4 Multiple treatments with one drug

Each Chinese herb usually has multiple effects and may play different roles in treating different diseases and prescriptions. The symptoms of patients with DKD are relatively complex, and doctors believe it is common to treat multiple symptoms with one Chinese herb during the TCM diagnosis and treatment of DKD. This study searched the question of “multiple treatments with one drug” using Cypher sentences. Taking “Poria” as an example. The specific sentences are as follows.

MATCH (c:`Chinese herb`)-[r]-(s:symptom).

WHERE c. name = ‘Poria’ RETURN c, r, s.

**FIGURE 9:**
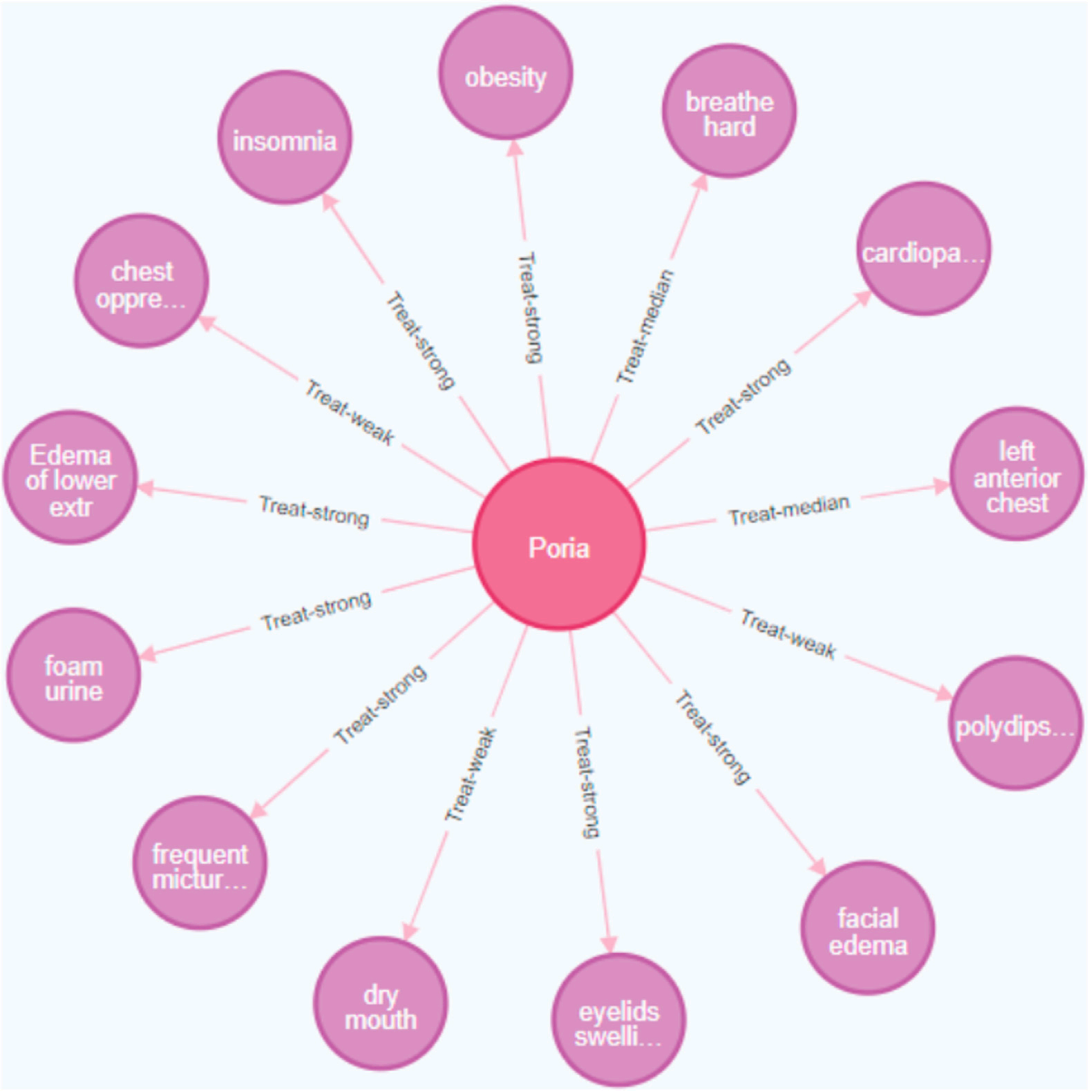
Knowledge discovery of “multiple treatments with one drug”. (The pink nodes represent Chines herbs, and the purple node represents the symptoms).

Through the association retrieval of the knowledge graph, it can be found that the same Chinese herb plays different roles in treating different symptoms, and the superior Chinese herbs for the symptom can also be found through the knowledge graph. For example, Poria can treat various symptoms such as edema, palpitation, insomnia, chest pain, foamy urine, dry mouth, etc. However, in the treatment of the dry mouth, Poria has a weak therapeutic effect, but a stronger therapeutic effect on edema and foam urine (Figure 9). We can quickly capture effective treatment plans through the knowledge graph and provide a reference for optimizing clinical diagnosis and treatment plans. After expert verification, the results are more consistent with clinical practice.

#### 3.4.5 Comorbidities may affect the choice of therapeutic method

In the process of clinical diagnosis and treatment of diseases, doctors usually choose the corresponding therapeutic method according to the patient’s symptoms. Analysis of the knowledge in the knowledge graph found that in the choice of treatment method, not only are the clinical symptoms and syndromes of patients considered, but the relevant medical history of comorbidities may also influence the treatment. This study used Cypher statements to query, and the details are as follows:

MATCH(d:Disease)-[r:`Coexist-strong`]->(d1:Disease), (d:Disease)-[r1:`Property of`]->(s:`Syndrome groups`)-[r2:Contains]->(s1:Syndrome) <-[r3:Treats] - (t:`Therapeutic method`).

WHERE d. name = 'Diabetic kidney disease’ and s. name = 'Syndrome groups of dampness-heat'.

RETURN d,d1,s,s1,t,r,r2,r3.

**FIGURE 10:**
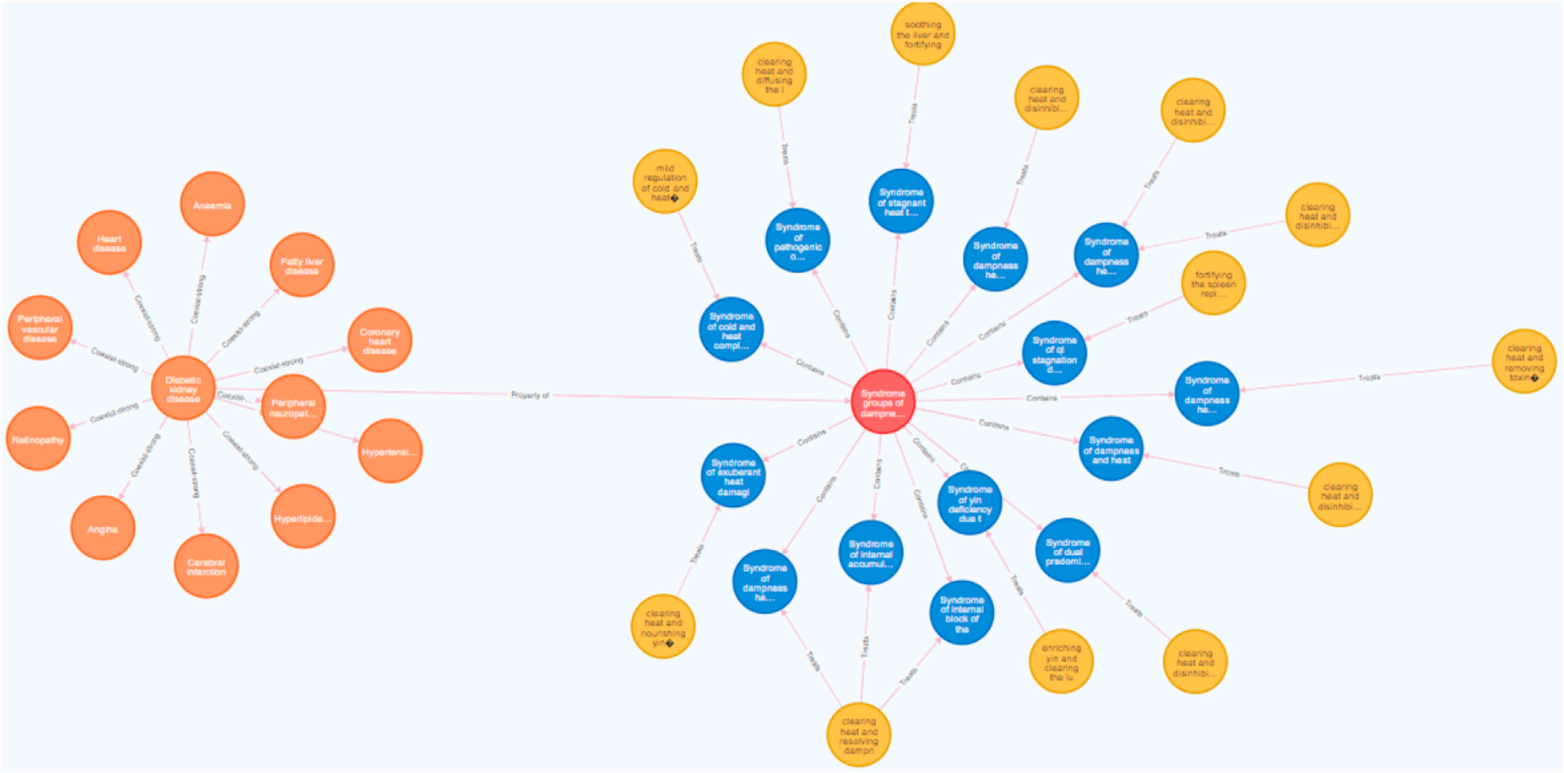
Comorbidities affect of the choice of therapeutic methods. (Orange nodes represent diseases (Diabetic kidney disease and comorbidities), the red node represents the syndrome group, blue nodes represent the syndromes, and yellow nodes represent therapeutic methods.).

The results showed the patient’s syndrome was diagnosed as a damp-heat syndrome, and the therapeutic method of tonifying the spleen and replenishing qi was chosen for treatment, which might be related to the patient’s complicated retinopathy, hypertension, and peripheral neuropathy (Figure 10). Experts believe that this performance improves the accuracy of diagnosis and treatment scheme recommendations. In the clinical diagnosis and treatment of DKD, comorbidities are critical links and are easily ignored. The graph can fully consider the influence of comorbidities, which is significant for improving the clinical efficacy of TCM diagnosis and treatment of DKD.

#### 3.4.6 The treatment plans for the same syndrome diagnosis can vary across different stages

The characteristic of TCM diagnosis and treatment for diabetic kidney disease is treatment based on stages. Fully understanding the differentiation of stages and syndromes is essential in diagnosing and treating DKD in TCM. In this study, retrieval analysis was conducted on the DKD knowledge graph, combining it with expert inquiries. The examples here are limited to nodes of DKD stages III and IV. The specific sentences are as follows:

MATCH (d:Disease)-[r:Process]->(s:Stage)

(s:Stage)-[r1]->(s1:`Syndrome groups`)-[r2:Contains]->(s2:Syndrome), (t:`Therapeutic method`)-[r3:Treats]->(s2:Syndrome), (t:`Therapeutic method`)-[r4]->(f:Formula)

WHERE s1. name = ‘Syndrome groups of Yang deficiency in spleen and kidney’ and s. name = ‘Phase III’ or s. name = ‘Phase IV’.

RETURN d,s,s1,s2,t,f,r,r1,r2,r3,r4.

**FIGURE 11:**
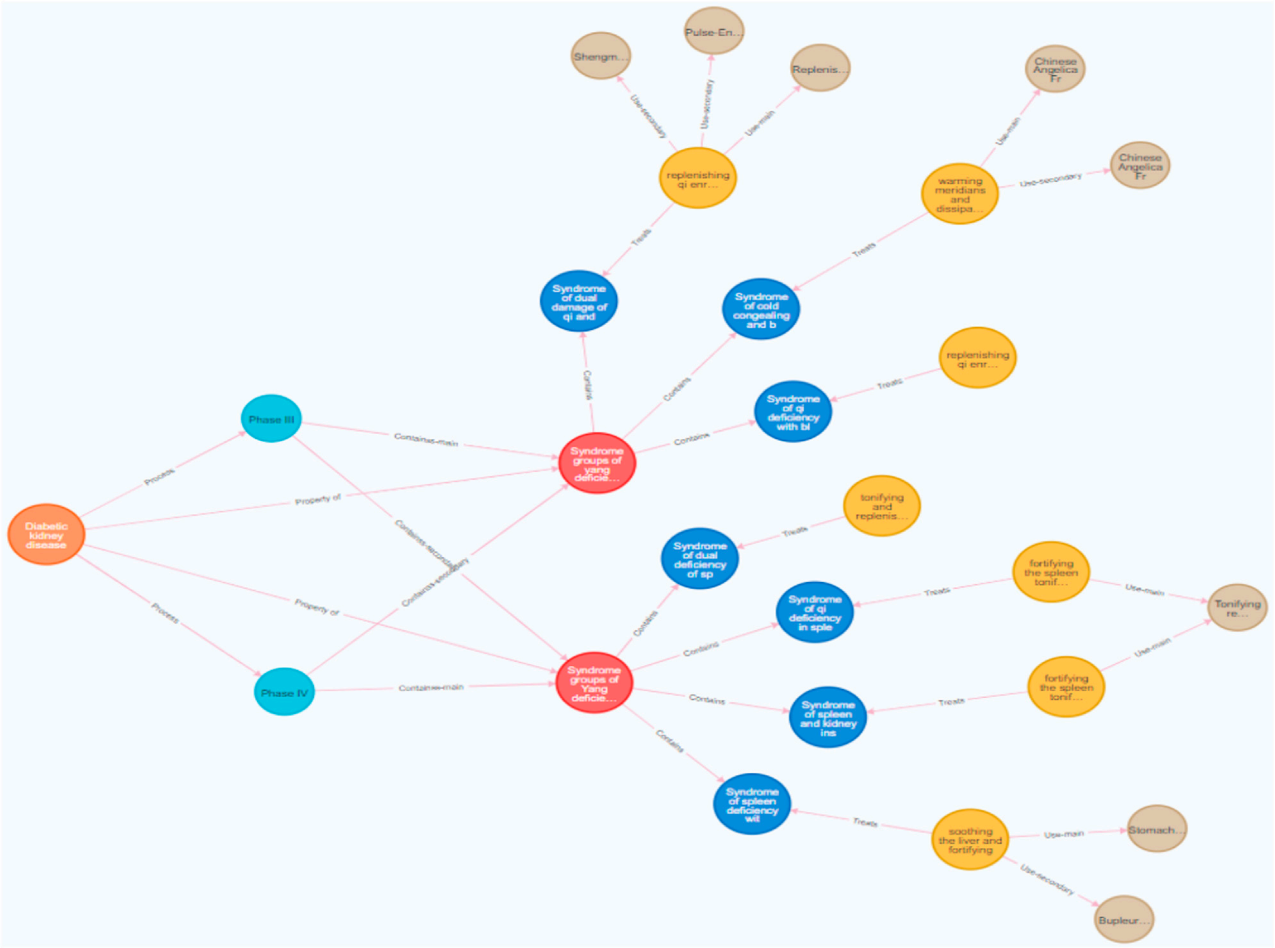
The influence of Different Stages and Syndromes of Diabetic kidney disease on the choice of TCM. (The orange node represent diseases (Diabetic kidney disease), light blue nodes represent stages, red node represent therapeutic metods and brown nodes represent formulae.).

The results showed that the stage might influence the choice of therapeutic formulae. For example, the syndrome of spleen and kidney yang deficiency is distributed in stages III and IV of DKD, with IV being the most common. The choice of formulae is also different in treating DKD with spleen-kidney yang deficiency. Spleen and kidney deficiency syndrome can be treated with Jianpi Yiqi Tiaotang Decoction or Shenqi Dihuang Decoction; The spleen and kidney yang deficiency syndrome can be treated with Linggui Zhugan Decoction. There are also corresponding differences in the selection of treatment options for different stages of the disease in the same syndrome group (Figure 11). According to the knowledge graph of TCM diagnosis and treatment for DKD, it is found that different stages may have a particular impact on the selection of basic treatment formulae for DKD.

## 4 Discussion

Clinical decision-making plays a vital role in the diagnosis and treatment process, as the development of clinical protocols directly impacts patients’ treatment outcomes and prognosis. Therefore, selecting the optimal treatment plan is of great significance. TCM emphasizes individualized syndrome differentiation and treatment, resulting in a relatively complex diagnosis and treatment process. The empirical knowledge of TCM practitioners is often accumulated through clinical practice, making it challenging to obtain effective diagnosis and treatment knowledge directly. Relying solely on medical guidelines may not address all clinical situations encountered. As an efficient and convenient knowledge management tool, a knowledge graph utilizes mathematical algorithms to construct knowledge structures and relationships. This approach enables more effective description and mining of relationships among entities, facilitating the efficient integration and management of TCM data resources (Li et al., 2020; Wang et al., 2022). By adopting a holistic perspective, knowledge graphs provide an effective and objective means to access the experience of TCM practitioners.

Currently, knowledge graphs are widely applied in the medical field. However, their application in the field of TCM is still relatively limited. Most research has focused on entity and relationship extraction methods, as well as the development of model algorithms (Qi et al., 2021; Jin et al., 2023). These research methods provide excellent technical support for discovering and reasoning TCM-related diagnosis and treatment knowledge. However, due to the lack of a standardized and comprehensive modeling strategy and process, they offer limited guidance for clinical application. In the field of TCM, research mainly involves organizing and exploring diagnosis and treatment knowledge found in ancient Chinese medical literature (Yu et al., 2017; Yang et al., 2021). There is relatively little research on implicit knowledge discovery in real-world TCM diagnosis and treatment data. This discrepancy between acquired knowledge and clinical reality may result in limited clinical guidance. Furthermore, most existing studies focus on general knowledge graphs (Long et al., 2019; Weng et al., 2022), with a relative lack of graph studies that incorporate clinical disease characteristics. The application of graphs aims to obtain more accurate and comprehensive knowledge of TCM diagnosis and treatment, screen effective diagnosis and treatment plans, and improve clinical efficacy. However, current research on TCM knowledge graphs has yet to yield a TCM knowledge system that integrates disease characteristics, ancient wisdom, and modern innovations.

This study focuses on the complexity of DKD and combines relevant literature, such as guidelines and consensus for TCM diagnosis and treatment of DKD, along with real-world clinical data. A knowledge graph of TCM diagnosis and treatment for DKD is then constructed, which incorporates disease characteristics, top-level wisdom, and aligns with clinical practice. Data mining is performed on the clinical data from TCM diagnosis and treatment of DKD to enhance the entities and relationships within the knowledge graph and include additional factors that may influence diagnosis and treatment decisions. By constructing a knowledge graph of DKD and exploring its tacit knowledge, a comprehensive and accurate understanding of DKD can be obtained from both a holistic and local perspective. This graph can assist junior doctors in enhancing their understanding of diseases, improving their diagnosis and treatment skills, and stimulating clinical thinking. Additionally, it provides methodological references for knowledge discovery in the diagnosis and treatment of specific diseases in TCM.

However, there are some limitations to this study. It only included DKD diagnosis and treatment data from a single tertiary referral hospital, resulting in limited types and quantity of data, and consequently, limited representativeness of mined tacit knowledge. Furthermore, due to these data limitations, the relationships included in the knowledge graph of DKD TCM diagnosis and treatment also have certain limitations. To address these limitations, future research should consider including DKD diagnosis and treatment data from multiple centers and regions, aiming to optimize the types of entities and relationships and uncover more tacit knowledge.

## 5 Conclusion

In this study, a knowledge graph of DKD TCM diagnosis and treatment was constructed by combining guidelines and real-world data. The association weights were obtained from data mining results, which enriched the relationship types of DKD knowledge graphs. The construction of the DKD knowledge graph not only realizes the systematic sorting of fragmented knowledge of DKD diagnosis and treatment but also provides an intuitive visualization of diagnosis and treatment knowledge. This study also explored tacit knowledge in the process of DKD diagnosis and treatment through the deep knowledge discovery of semantic retrieval, and realized the discovery and sharing of knowledge surrounding diabetic kidney disease diagnosis and treatment. The results of the study are of great significance in enhancing awareness of DKD TCM diagnosis and treatment knowledge among junior doctors. It provides valuable insights for supporting the development and refinement of TCM clinical diagnosis and treatment plans for DKD and serves as a foundation for building an intelligent diagnosis and treatment recommendation system for DKD.

## Data Availability

The original contributions presented in the study are included in the article/supplementary material, further inquiries can be directed to the corresponding authors.

## References

[B1] American Diabetes Association (2020). 2. Classification and diagnosis of diabetes: Standards of medical care in diabetes-2020. Diabetes Care 43 (1), S14–S31. 10.2337/dc20-S002 31862745

[B2] Cheng C. Zheng F. Hang H. (2013). Thoughts on development of electronic medical records system. China Digit. Med. 8 (8), 67–69. 10.3969/j.issn.1673-7571

[B3] Committee for the examination and approval of terms in traditional Chinese medicine (2005). Terms of traditional Chinese medicine. Beijing: Science Press.

[B4] Dongzhimen Hospital of Beijing University of Traditional Chinese Medicine (2017). Traditional Chinese medicine treatment plan for consumptive thirst involving kidney (diabetic kidney disease) (2017 Version). Chin. Pract. J. Rural Dr. 24 (8), 3. 10.3969/j.issn.1672-7185.2017.08.030

[B5] Gao Y. Liu T. Nan Z. Zhen Z. Zhou Q. (2011). TCM diagnosis and treatment standard of diabetic kidney disease. World J. Integr. Traditional West. Med. 6 (6), 548–552. 10.13935/j.cnki.sjzx.2011.06.016

[B6] Gong F. Ma Y. Gong W. Li X. Li C. Yuan X. (2018). Neo4j graph database realizes efficient storage performance of oilfield ontology. PloS One 13 (11), e0207595. 10.1371/journal.pone.0207595 30444913 PMC6239324

[B7] Guarino N. Oberle D. Staab S. (2009). “What is an ontology?,” in Handbook on ontologies, 1–17. 10.1007/978-3-540-92673-3_0

[B8] Huang Z. Hu Q. Liao M. Miao C. Wang C. Liu G. (2021). Knowledge graphs of kawasaki disease. Health Inf. Sci. Syst. 9 (1), 11. 10.1007/s13755-020-00130-8 33680447 PMC7910781

[B9] ICD-11 (2023). ICD-11. Available at: https://icd.who.int/zh (Accessed January 18, 2023).

[B10] ISO (2014). ISO/TS 17938. Available at: https://www.iso.org/standard/61071.html (Accessed January 18, 2023).

[B11] Jin Y. Ji W. Shi Y. Wang X. &Yang X. (2023). Meta-path guided graph attention network for explainable herb recommendation. Health Inf. Sci. Syst. 11 (1), 5. 10.1007/s13755-022-00207-6 36660407 PMC9847457

[B12] Kurbatova N. Swiers R. (2021). Disease ontologies for knowledge graphs. BMC Bioinforma. 22 (1), 377. 10.1186/s12859-021-04173-w PMC8296689 34289807

[B13] Li G. Wang H. Liu P. He H. Che X. Zhang H. (2019). TCM syndromes and their distribution regulatity in patients with diabetic nephropathy at stageⅢ,stage Ⅳ and stage Ⅴ. Hebei Med. J. 41 (7), 1054–1057. 10.3969/j.issn.1002-7386.2019.07.023

[B14] Li J. Ma L. (2015). Terminology specification for common clinical symptoms of TCM. Beijing: Medical Science and Technology Publishing House.

[B15] Li L. Wang P. Yan J. Wang Y. Li S. Jiang J. (2020). Real-world data medical knowledge graph: Construction and applications. Artif. Intell. Med. 103, 101817. 10.1016/j.artmed.2020.101817 32143785

[B16] Li L. Zhao J. Zhang Y. (2020). A lightweight airborne remote interface unit analogue acquisition BIT circuit design. C*hangjiang Inf. Commun.* (8), 83–85.

[B17] Li X. Liu H. Zhao X. Zhang G. Xing C. (2020). Automatic approach for constructing a knowledge graph of knee osteoarthritis in Chinese. Health Inf. Sci. Syst. 8 (1), 12. 10.1007/s13755-020-0102-4 32175080 PMC7046853

[B18] Li Y. Qi X. Chen H. (2016). Analysis on national famous old doctor of traditional Chinese medicine on diabetic nephropathy syndrome differentiation and its treatment rules. Med. Res. Educ. 33 (5), 13–20. 10.3969/j.issn.1674-490X

[B19] Li Z. Zhong Q. Yang J. Duan Y. Wang W. Wu C. (2022). DeepKG: An end-to-end deep learning-based workflow for biomedical knowledge graph extraction, optimization and applications. Bioinforma. Oxf. Engl. 38 (5), 1477–1479. 10.1093/bioinformatics/btab767 PMC8689937 34788369

[B20] Liang S. Cai G. Chen X. (2017). Clinical and pathological factors associated with progression of diabetic nephropathy. Nephrol. Carlt. Vic.) 22 (4). 10.1111/nep.13182 29155497

[B21] Liao J. (2007). Research on information retrieval based on domain ontology. Central south university. 10.7666/d.y1084589

[B22] Lin Y. Zhang F. Lei L. Wang P. Kang L. Ren J. (2020). Analysis on medication laws for TCM treatment of Diabetic Kidney Disease based on data mining. Chin. J. Inf. Traditional Chin. Med. 27 (5), 102–106. 10.3969/j.issn.1005-5304.201902169

[B23] Long H. Zhu Y. Jia L. Gao B. Liu J. Liu L. (2019). An ontological framework for the formalization, organization and usage of TCM-Knowledge. BMC Med. Inf. Decis. Mak. 19 (2), 53. 10.1186/s12911-019-0760-9 PMC6454592 30961578

[B24] Ma J. Li D. Chen Y. Qiao Y. Zhu H. Zhang X. (2021). A knowledge graph entity disambiguation method based on entity-relationship embedding and graph structure embedding. Comput. Intell. Neurosci. 2021, 2878189. 10.1155/2021/2878189 34603428 PMC8486511

[B25] Microvascular Complications Group of the Chinese Medical Association Diabetes Branch (2021). Guidelines for the prevention and treatment of diabetic kidney disease in China (2021 Version). Chin. J. Diabetes 13 (8), 23.

[B26] National Health Commission, State Administration of traditional Chinese medicine (2020a). Clinic terminology of traditional Chinese medical diagnosis and treatment—Part 2:syndromes.

[B27] National Health Commission, State Administration of traditional Chinese medicine (2020b). Clinic terminology of traditional Chinese medical diagnosis and treatment—Part 3: Therapeutic methods.

[B28] National Pharmacopoeia Committee (2020). Pharmacopoeia of the people’s Republic of China (2020 version). Beijing: China medical science press.

[B29] Qi T. Qiu S. Shen X. Chen H. Yang S. Wen H. (2021). KeMRE: Knowledge-enhanced medical relation extraction for Chinese medicine instructions. J. Biomed. Inf. 120. 10.1016/j.jbi.2021.103834 34119692

[B30] Rossanez A. Dos Reis J. C. Torres R. D. S. de Ribaupierre H. (2020). KGen: A knowledge graph generator from biomedical scientific literature. BMC Med. Inf. Decis. Mak. 20 (4), 314. 10.1186/s12911-020-01341-5 PMC7734730 33317512

[B31] Santos A. Colaço A. Nielsen A. Niu L. Strauss M. Geyer P. E. (2022). A knowledge graph to interpret clinical proteomics data. Nat. Biotechnol. 40 (5), 692–702. 10.1038/s41587-021-01145-6 35102292 PMC9110295

[B32] Singhal A. (2012). Introducing the knowledge graph: Things, not strings. Available at: https://blog.google/products/search/introducing-knowledge-graph-things-not/ .

[B33] United States Renal Data System | USRDS | NIDDK (2023). National Institute of diabetes and digestive and kidney diseases. Available at: https://www.niddk.nih.gov/about-niddk/strategic-plans-reports/usrds (Accessed January 18, 2023).

[B34] Wang H. Qi G. Chen H. (2019). Knowledge graph method, practice and application. Beijing: Publishing House of Electronics Industry.

[B35] Wang S. Li Z. Yang T. Hu K. (2022). Current status and development trend of knowledge graph research in traditional Chinese medicine. J. Nanjing Univ. Traditional Chin. Med. 38 (3). 10.14148/j.issn.1672-0482.2022.0272

[B36] Wang Y. Zhao J. Wang S. Pang B. Li J. Zhang Y. (2014). Research on TCM syndrome evolution of diabetic kidney disease on the basis of factor analysis and transition probability analysis. J. Traditional Chin. Med. 55 (15), 1317–1322. 10.13288/j.11-2166/r.2014.15.016

[B37] Weng H. Chen J. Ou A. Lao Y. (2022). Leveraging representation learning for the construction and application of a knowledge graph for traditional Chinese medicine: Framework development study. JMIR Med. Inf. 10 (9), e38414. 10.2196/38414 PMC9482071 36053574

[B38] Xiao R. Hu F. Pei W. Bie M. (2021). Research on data mining model of group prescription based on the basic theory of TCM. Lishizhen Med. Materia Medica Res. 32 (1), 254–256.

[B39] Yang N. Liu X. (2007). Diagnosis, syndrome differentiation and curative effect evaluation criteria of diabetes nephropathy (Trial Scheme). Shanghai J. Traditional Chin. Med. 7, 7–8. 10.16305/j.1007-1334.2007.07.003

[B40] Yang X. Wu C. Nenadic G. Wang W. Lu K. (2021). Mining a stroke knowledge graph from literature. BMC Bioinforma. 22 (10), 387. 10.1186/s12859-021-04292-4 PMC8319697 34325669

[B41] Yao N. (2005). Differential diagnosis of symptoms in TCM. Beijing: People's Medical Publishing House.

[B42] Yu J. Ni Q. Liu S. (2022). Guidelines for diagnosis and treatment of diabetic kidney disease with disease and syndrome. J. Traditional Chin. Med. 63 (2), 190–197. 10.13288/j.11-2166/r.2022.02.018

[B43] Yu T. Li J. Yu Q. Tian Y. Shun X. Xu L. (2017). Knowledge graph for TCM health preservation: Design, construction, and applications. Artif. Intell. Med. 77, 48–52. 10.1016/j.artmed.2017.04.001 28545611

[B44] Zhang J. Liu Z. Xie H. Ge Y. Zhang P. Li L. (2009). Chronic complications in patients of type 2 diabetes with diabetic nephropathy. Chin. J. Nephrology,Dialysis Transplant. 18 (3), 218–225. 10.3969/j.issn.1006-298X.2009.03.004

[B45] Zhao J. Wang S. Li J. Huang W. (2017). Studies about specification of syndrome differentiation on different stages and efficacy evaluation proposal for diabetic kidney disease. World Chin. Med. 12 (1), 1–4.

[B46] Zhu Q. Nguyen D. Grishagin I. Southall N. Sid E. Pariser A. (2020). An integrative knowledge graph for rare diseases, derived from the Genetic and Rare Diseases Information Center (GARD). J. Biomed. Semant. 11 (1), 13. 10.1186/s13326-020-00232-y PMC7663894 33183351

[B47] Zhu W. (2008). Syndrome element syndrome differentiation (SESD). Beijing: People’s Health Publishing House.

